# Meckel's diverticulum axial torsion: A rare complication case report of a 5-year-old girl

**DOI:** 10.1016/j.ijscr.2023.107883

**Published:** 2023-01-07

**Authors:** Ramin Kafshgari, Amirmohamad Rezaei Majd, Ali Taherinezhad Ledari

**Affiliations:** a Assistant Professor of Pediatric Surgery, Department of Pediatrics, Amirkola Hospital, Babol University of Medical Sciences, Babol, Iran; b Student Research Committee, Babol University of Medical Sciences, Babol, Iran

**Keywords:** Meckel's diverticulum, Meckel's torsion, Axial torsion, Pediatric surgery, Case report

## Abstract

**Introduction:**

Meckel's diverticulum is the most common congenital malformation of the gastrointestinal tract. Axial torsion of the diverticulum followed by gangrene is the rarest complication that can occur mainly in children.

**Case presentation:**

A 5-year-old girl complaining of vomiting, fever and abdominal pain came to hospital. In Laboratory findings, leukocytosis (WBC = 22.5 ∗ 10^3^/μl) was observed and mild interloop fluid with a whirlpool-like structure which suggests volvulus like obstruction was seen in sonography. The patient underwent emergency laparotomy. A necrotic congested Meckel's diverticulum was found during the surgery, which was axially twisted. After the operation, the patient recovered and was discharged six days later.

**Discussion:**

Axial twisting of Meckel's diverticulum is known as one of the rarest related complications, and it is caused by the rotation of the diverticulum around its axis. Among the factors that can make Meckel's diverticulum prone to twisting are its connection to the intestinal mesentery or the umbilical cord, or the presence of mesodiverticular bands. One of the appropriate diagnostic methods is the use of technetium-99. In Cases of small bowel obstruction, diverticulectomy and segmental or wedge resection have been introduced as suitable surgical methods. The delay in diagnosing a complex Meckel's diverticulum can lead to complications and mortality.

**Conclusion:**

Rapid management of Meckel's diverticulum, which has become challenging due to its difficult diagnosis, is very important to obtain acceptable results.

## Highlights

• Axial twisting of Meckel's diverticulum is known as one of the rarest related complications.
• Its connection to the intestinal mesentery or the umbilical cord is potential risk factors.
• Delay in diagnosing a complex Meckel's diverticulum can lead to complications and mortality.
• Rapid management of Meckel's diverticulum, is very important to obtain acceptable results.

## 1 Introduction

Meckel's diverticulum, first described by Johann Friedrich Meckel in 1809 of its fetal origin, is the most common congenital malformation of the gastrointestinal tract that occurs in 2 % of the population [1], [2]. This diverticulum is caused by incomplete obliteration of the vitelline or omphalomesenteric duct, which occurs in the fifth to seventh weeks of fetal development [3]. The location of this diverticulum is usually 40 to 50 cm proximal to the ileocecal valve although its size varies, but it is usually 2 in. long and 2 cm in diameter [2], [4]. People who suffer from this diverticulum are primarily asymptomatic, and only 2 % of cases are symptomatic [2]. The symptoms that occur in these people can be caused by bleeding and intestinal obstruction, which are among the most complications of Meckel's diverticulum and occur more commonly in children [5].

Other complications of Meckel's diverticulitis which is most common in adults include diverticulitis, perforation, Littre's hernia, and tumors [6]. Axial torsion of the diverticulum followed by gangrene is the rarest complication that can occur, mainly in children [5]. Twisting of the diverticulum takes place with the help of two omphalomesenteric and mesodiverticular bands, omphalomesenteric band is the remaining of the omphalomesenteric duct and the mesodiverticular band is the remaining of the embryonic vitelline blood circulation. These two bands establishes an axis point for diverticular torsion, and due to the axial rotation of the diverticulum around its narrow base, blood supply and venous return will be disrupted and finally gangrene will occur [7]. In this case report we introduce and discuss about this rare condition that could be difficult to diagnose. This case report has been reported in line with the SCARE criteria [8].

## 2 Case presentation

A 5-year-old girl came to the emergency room complaining of vomiting and abdominal pain. The patient's vomit contains food, and after continuous vomiting it became green and contains bile. The abdominal pain started simultaneously with the vomiting, and it was in the periumbilical region. The patient had defecation previous day. Also, the patient had mentioned a history of constipation, but she had not received any medication for that and had no history of surgery. In physical examination vital signs were *T* = 38.9, RR = 18, PR = 100, BP = 100/70 mmHg. In abdominal examination a mild distension was seen and patient had guarding and tenderness in periumbilical and right lower quadrant area, in palpation fecal mass was also felt. In Laboratory findings, leukocytosis (WBC = 22.5 ∗ 10^3^/μl) was observed, which consisted of 89 % neutrophils, 9 % lymphocytes, and 1 % eosinophils. Other laboratory tests were within normal ranges. Abdominal and pelvic ultrasound was performed for the patient, during that a dilated lobe with a prominent wall, along with a transitional zone and increased fat echogenicity was seen in the RLQ and a mild interloop fluid with a whirlpool-like structure which suggests volvulus like obstruction. Also, a normal and non-inflamed appendix was seen.

**Figs. 1 & 2:**
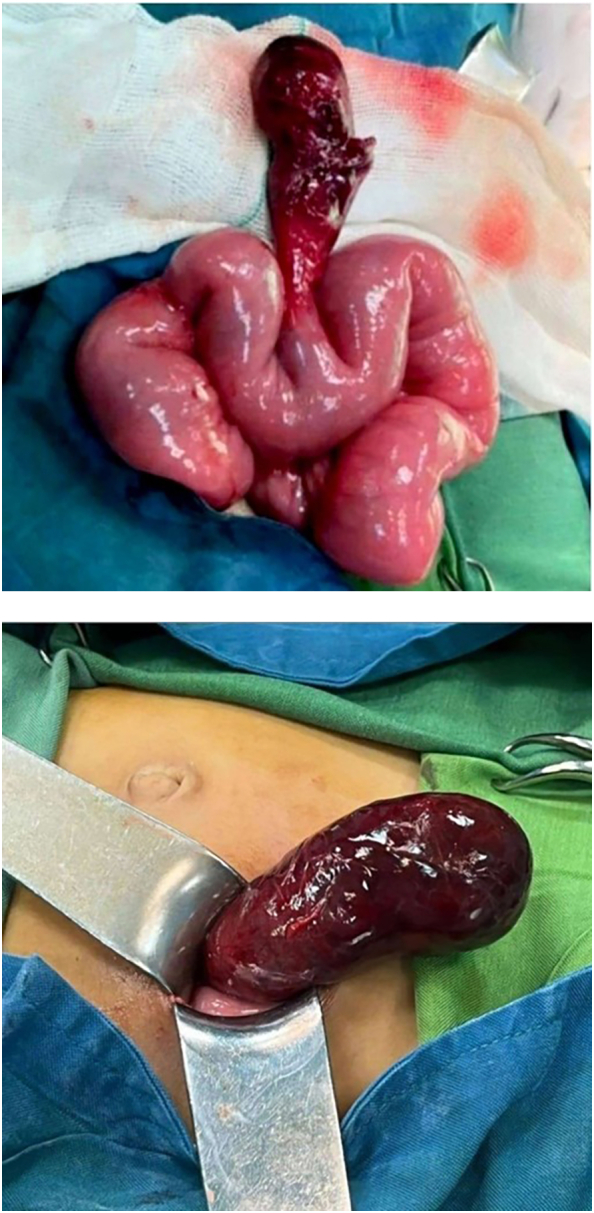
A necrotic congested Meckel's diverticulum was found during the surgery, which was axially twisted and led to bowel obstruction.

These findings raised the diagnosis of obstructive processes in the field of volvulus. The patient underwent emergency laparotomy after adequate hydration. During the operation, a small amount of hemorrhagic fluid was observed in the peritoneal cavity along with dilated loops of the small intestine. A necrotic congested Meckel's diverticulum was found during the surgery, which was axially twisted and led to bowel obstruction (Fig. 1). Approximately 20 cm of the small intestine was resected along with Meckel's diverticulum and anastomosed. Also, appendectomy was done for the patient. After the operation, the patient recovered and discharged six days later.

## 3 Discussion

Meckel's diverticulum, which is known as the most common abnormality of the GI (gastrointestinal)system, consists of all the layers of the GI system, including mucous, muscular and serous, which turns it into a true diverticulum based on the definition [1], [9], [10]. The remnant of the vitelline duct, which naturally recedes during the 6-8th fetal week, may sometimes remain in the form of a blind ring in the distal ileum and form this diverticulum [10], [11].

The incidence of Meckel's diverticulum is generally higher in males, and based on the studies, it has an incidence rate between 0.3 and 2.9 % [12]. Meckel's diverticulum usually occurs 90 cm from the ileocecal valve in 90 % of the cases. However, some cases had been reported that occurred at a distance of 180 cm from this valve [3].

Although the size of this diverticulum is variable, its usual size is 2.9 cm in length and 1.9 cm in width [3].

This diverticulum doesn't cause any symptoms except in 4 to 6 % of cases. The symptoms that occur in people also include common symptoms such as abdominal pain that can occur with or without fever, as well as vomiting or constipation, rectal bleeding, abdominal distension, and also in rare cases, chronic pain. These symptoms can, be different according to the complication that arose after it [13], [14]. As in our case, such symptoms were also observed, which made Meckel's diverticulum one of our differential diagnosis. The occurrence of complications related to this diverticulum has the same distribution among all ages, however, it has been reported to have a higher incidence in males, which can be a reason for identifying more cases of this diverticulum in them [3], [11]. 40 % of these complications are attributed to intestinal obstruction, which is known as the most common complication of Meckel's diverticulum, followed by peritonitis, which accounts for 5 to 19 % of cases [15], [16].In a previous cohort study about Meckel's diverticulum in pediatrics most patients diagnosed at the age of 3 to 15 years, like in our case. However, the most common symptom was rectal bleeding without abdominal pain, but also intestinal obstruction was seen mostly because of Meckel's band and adhesive changes in diverticulitis [16].

Axial twisting of Meckel's diverticulum is known as one of the rarest related complications, and it is caused by the rotation of the diverticulum around its axis, without involving the connected ileal ring or ileal mesentery, which in this way may lead to gangrene by disrupting the process of blood supply to its tissue [17]. Among the factors that can make Meckel's diverticulum prone to twisting are its connection to the intestinal mesentery or the umbilical cord, or the company of mesodiverticular bands, and the greater length and size of the diverticulum or its narrow base are among the predisposing factors [7]. Abdominal pain in Right lower quadrant region, which can last for a different period, is one of the common symptoms related to torsion [17], [18]. According to the existing non-specific symptoms, its diagnosis may be delayed and items such as appendicitis, small bowel obstruction, cholecystitis, and amoebic liver abscess are considered. In the case of gangrene of the twisted tissue or its perforation, there is a possibility of peritonitis and sepsis, which makes time of diagnosis so vital [17].

There are several imaging methods that can be used for this diverticulum, such as X-ray, which can detect intestinal obstruction and pneumoperitoneum with the presence of enteroliths, or gas-filled diverticulum but it has a low diagnostic value. Another method is CT scan that can show intestines full of gas, although it is challenging to distinguish Meckel's diverticulum from a normal intestine in this method [16], [19]. One of the appropriate diagnostic methods for this is the use of technetium-99 m, which absorbed by the heterotopic mucus of the stomach and can be very helpful in diagnosis [16]. In this case; we only relied on ultrasound, and considering the need for laparotomy by physical exam and the deterioration of the patient's condition, we did not waste time.

Performing surgery is a suitable treatment method for Meckel's diverticulum, both laparoscopy and laparotomy have achieved very good results [20]. In cases of small bowel obstruction by diverticulum, diverticulectomy, segmental or wedge resection have been introduced as suitable surgical methods. The wedge resection method preferred due to avoiding the dissection of the mesentery, and intestinal anastomosis, as well as shorter surgical time and lower complications, especially in cases where the base of the diverticulum is intact [12].

In general, the results of Meckel's diverticulum operations, as seen in this case are good. Still, the delay in diagnosing a complex Meckel's diverticulum can lead to complications and mortality.

## 4 Conclusion

Torsion of Meckel's diverticulum is a rare complication of this disease that can lead to acute abdomen. The risk of twisting can increase due to factors such as increasing the length and narrowness of its base. This complication could easily mistake with appendicitis and other diseases because of its non-specific symptoms. And due to inappropriate diagnostic methods, its diagnosis is associated with great uncertainty. Rapid management of Meckel's diverticulum, which has become challenging due to its difficult diagnosis, is very essential to obtain acceptable results.

## Consent

Written informed consent was obtained from the patient's parents/legal guardian for publication of this case report and accompanying images. A copy of the written consent is available for review by the Editor-in-Chief of this journal on request.

## Ethical approval

The authors institute provided ethical approval for this case study.

## Sources of funding

N/A.

## Author contribution

Ramin Kafshgari: Did the Surgery, lead the Study.

Amirmohamad Rezaei majd: Collected Data, Revised Manuscript.

Ali Taherinezhad Ledari: Collected Data, Wrote Manuscript.

## Guarantor

Ali Taherinezhad Ledari.

## Research registration

N/A.

## Declaration of competing interest

N/A.
